# Down regulation of the *PEDF* gene in human lens epithelium cells changed the expression of proteins vimentin and αB-crystallin

**Published:** 2010-01-26

**Authors:** Jing Yang, Lixia Luo, Xialin Liu, Mark I. Rosenblatt, Bo Qu, Yuhua Liu, Yizhi Liu

**Affiliations:** 1 State Key Laboratory of Ophthalmology, Zhongshan Ophthalmic Center, Sun Yat-sen University, Guangzhou, China; 2 The Margaret M. Dyson Vision Research Institute, Weill Cornell Medical College, New York, NY; 3 Department of Ophthalmology, Weill Cornell Medical College, New York, NY

## Abstract

**Purpose:**

To study the relationship of pigment epithelium-derived factor (*PEDF*) expression with the expression of vimentin and αB-crystallin by lens epithelial cells.

**Methods:**

Lens epithelial cells adhering to anterior capsules taken from young donor eyes aged from 20 to 35 years were cultured and passaged. We designed small interfering RNA (siRNA) constructs to specifically downregulate the expression of *PEDF* by these primary lens epithelial cells. Quantitative PCR was used to confirm the downregulation of *PEDF* RNA expression following infection of lens epithelial cells. To determine whether altering the expression of *PEDF* would effect the expression of vimentin or αB-crystallin, we performed western blotting 48 h after expression of the *PEDF*-directed siRNA.

**Results:**

*PEDF* RNA expression in the human lens epithelial cells was strongly downregulated by the three separate siRNA constructs. Western blotting revealed that the downregulation of *PEDF* expression resulted in a concomitant decrease in expression of vimentin and an increase in αB-crystallin protein.

**Conclusions:**

Decreased expression of *PEDF* in primary human lens epithelial cells resulted in a decrease in the expression of vimentin and the increase of αB-crystallin expression, two proteins critical for maintaining lens clarity.

## Introduction

Pigment epithelium-derived factor (PEDF), a 50-kDa secreted protein, is a member of the serpin family of proteins and is expressed in all ocular tissues of the human eye [1]. PEDF accumulates in the aqueous humor [2]. It acts as a survival factor, protecting neuronal cells from natural and induced apoptosis [3]. Lens function deteriorates with age. Oxidative stress, ultraviolet radiation, and other toxic factors can induce the formation of cataract in vitro and in vivo [4-6]. The formation of senile cataract is a universal aging process accompanied by numerous morphological and functional changes in the lens cells. Apoptosis of lens epithelial cells appears to be a common cellular basis for noncongenital cataract (including senile) development in humans and animals [7]. PEDF is expressed intracellularly in almost all human ocular tissues and extracellularly during both fetal and early adult periods [1-3,8].

Our previous study found that the *PEDF* gene can be expressed in aqueous humor and lens epithelial cells, and that the expression level decreased significantly with increasing age. Those results suggested *PEDF* may regulate or protect the lens epithelial cells by paracrine or autocine processes [9].

Additionally, one study of the molecular characteristics of lens epithelial cells from patients with senile cataract by cDNA microarray technique found that *PEDF* was strongly downregulated (by 5.9-fold) in senile cataract [9]. The *PEDF* gene is known to have an important role in the physiology and morphology of the transparent lens [10]. As we know, the opacification of eye lens is often caused by protein misfolding and aggregation. There may, therefore, be some relationship between *PEDF* and some lens proteins, such as αB-crystallin and vimentin. However, there is little available information on this, so we designed our experiment to determine whether downregulation of *PEDF* expression is associated with alterations in the expression of the critical lens proteins vimentin and αB-crystallin.

The reason we chose αB-crystallin and vimentin to study is that these are important proteins for lens. αB-crystallin, a member of the small heat shock protein family is among the predominant proteins of the vertebrate eye lens and is constitutively expressed at low levels in the lens epithelium and in numerous tissues [11-14]. Many factors are known to play a role in the formation of aggregated and cross-linked crystallin species during cataract development [15].

αB-crystallin is a major lens protein expressed in numerous nonlens tissues of vertebrates, consistent with its putative no-refractive cellular functions [12,16-19]. Patients who carry a missense mutation in aB-crystallin (R120G) develop desmin-related myopathy and cataracts [20]. It is likely that maintaining genomic integrity is important in the lens since the anterior lens epithelial cells are held in the G_0_ phase of the cell cycle throughout life [21,22]. One study suggested that aB-crystallin may be an important component of the cellular machinery involved in maintaining genomic stability [23]. The reduced thermal stability and the dominant negative effects of the mutant αB-crystallin may be the direct cause of cataract because αB-crystallin null mice have clear lenses [24].

Vimentin, a critical cytoskeletal element in the human lens cell, is a main structural determinant in these cells, forming a membrane-connected cytoskeleton. Vimentin shows a unique pattern of expression relative to all known intermediate filament (IF) proteins [25]. Vimentin is mainly expressed in the epithelium of the lens [26]. A previous study revealed that high expression of vimentin interfered strongly with the normal differentiation of the lens fibers. Normal fiber cell denucleation and elongation processes were impaired and the animals developed pronounced cataracts followed by extensive lens degeneration. The age of appearance and extent of these abnormalities in the different transgenic lines were directly related to the vimentin level [26]. Overexpression of this protein has strongly disturbed the normal morphogenesis of the lens [26]. Decrease of vimentin may be initiated by damage to the epithelial cells, leading to degradation of the cytoskeleton, and appears to be related to the formation of age-related cataract [27].

Through this experiment, we found a significant overexpression of αB-crystallin and downexpression of vimentin in human primary lens epithelial cells through RNAinterference (RNAi) RNAi to PEDF.

## Methods

### Source of eye tissue

Human eyes were obtained from cadavers (12 female, 15 male, aged from 20-35 years) through the ZhongShan Ophthalmic center eye Bank, Guangzhou, China. Investigations that involved only residual material from autopsy were approved by the Human Subjects Committee of Sun-yet-Sen University, Guangzhou, China. We certify that all applicable institutional and governmental regulations concerning the ethical use of human volunteers/animals were followed during this research.

### Human epithelial cells primary culture

Lens epithelial cells adhering to anterior capsules were harvested from cadaver lenses. Under a dissecting microscope, lens capsules were spread onto the tissue culture dishes, epithelium side up. A single drop of fetal bovine serum was placed on the epithelial surface to prevent desiccation. Incubation for 2 h at 37 °C and 5% CO_2_ was performed to allow for adequate adherence of the tissue to the dish. Dulbecco’s modified Eagle’s medium (DMEM) made to 20% fetal bovine serum was then added, and the cells were incubated under the same conditions until further experimentation.

### Primary human lens epithelial cell infection

We designed three small hairpin RNA (shRNA) sequences (sample 2, 3, and 4) directed against human *PEDF* and scramble sequence (sample 1), using GenScript’s small interfering RNA (siRNA) design center, siRNA Target Finder, and siRNA Construct Builder (Table 1). Oligonucleotides corresponding to these sequences were cloned using standard molecular biology techniques into plasmid vector (SD1211) for transient transfection experiments and into pLenti6/V5-D-TOPO for generation of lentiviral shRNA particles.

**Table 1:** shRNA sequences for different PEDF RNAi groups.

**Sample**	** Antisense | Loop | Sense**
Sample1 (scramble)	CGGTGATAATTCTAATGGCGTTTGATATCCGACGCCATTAGAATTATCACCG
Sample2 (siRNA insert 1)	TCTTGCAGTTGAGATCAGAGTTTGATATCCGACTCTGATCTCAACTGCAAGA
Sample3 (siRNA insert 2)	TCTCAGGCGGTACAGATCGTATTGATATCCGTACGATCTGTACCGCCTGAGA
Sample4 (siRNA insert 3)	TAAGCCACGCCAAGGAGAAGGTTGATATCCGCCTTCTCCTTGGCGTGGCTTA

Using the reagents provided in the ViraPower™ Lentiviral Expression Systems (Invitrogen, Guangzhou, China), the ViraPower™ packaging mix and a control lentiviral construct were cotransfected into 293FT cells using the protocol provided by the company. Lentiviral supernatants were harvested 48 h posttransfection.

The day before transfection, 293FT cells were plated in a 10-cm tissue culture plate. On the day of transfection, the culture medium was removed from the 293FT cells and was replaced with 5 ml of Opti-MEM® I medium (GIBCO, invitrogen Guangzhou, China). DNA-Lipofectamine™ 2000 (Invitrogen, Guangzhou, China) complexes were added dropwise to each plate of cells and gently mixed. The cells were incubated overnight at 37 °C in a humidified 5% CO_2_ incubator. The next day, the medium containing the DNA-Lipofectamine™ 2000 complexes was removed and replaced with 10 ml complete culture medium without antibiotics. The cells were incubated overnight at 37 °C in a humidified 5% CO_2_ incubator. The virus-containing supernatants were harvested 48–72 h posttransfection.

Serial diluted vector stock and 4 μl/ml polybrene (Sigma-Aldrich, Invitrogen Guangzhou, China) was added to the cultured primary human lens epithelial cells. This was followed by overnight culture at 37 °C in 5% CO_2_, and the culture medium was then replaced. Culturing was continued for 48 h.

### RNA extraction

Total RNA was extracted with RNeasy plus Mini kit (Qiagen, Valencia, CA) from the primary human lens epithelial cells 2 days after infection with shRNA expression vectors. Protocol is as follows: Havest the cells, then disrupt the cells with buffer RLT (in kit), add 70℅ ethanol, mix and transfer the mixture into RNEASY spin column, vortex 2 min at 13,000 rpm. Then wash the column with buffer RW and buffer RPE. Finally elute the RNA with 50 µl RNease-free water. All the reagents were used in this step are in the kit. RNA quality was assessed by running 0.5–1 µg of RNA on 1% agarose gels. Samples that were found to contain degraded RNA were discarded. RNA concentrations were measured by absorption at 260 nm (NanoDrop™ 2000 spectrophotometer from Thermo Scientific, St. Louis, MO). RNA samples were made to the same concentration prior to cDNA synthesis using the High Capacity cDNA Reverse Transcription Kit (Applied Biosystems, Invitrogen, Guangzhou, China). RT Buffer (10×, 2.0 μl), 25× dNTP Mix (0.8 μl), 10× RT Random Primers (1.0 μl), MultiScribe™ Reverse Transcriptase (1.0 μl), RNase Inhibitor (1.0 μl), Nuclease-free H2O (3.2 μl), and RNA sample (10 μl) were mixed briefly and vortexed. Then the cocktail was allowed to react at 25 °C for 10 min, then 37 °C for 120 min, then 85 °C for 5 s, then keep at 4 °C. The resultant cDNA for real time PCR a StepOne™ Real-Time PCR System from Applied Biosystems instrument using the Power SYBR Green PCR Master Mix (Applied Biosystems , Invitrogen, Guanghzou, China). For each well, we mixed 6 μl 2× Power SYBR Green Master Mix, 0.5 μl forward primer, 0.5 μl reverse primer, and 5 μl CDNA sample. Polymerase activation were performed at 95 °C for 10 min, then the reaction were performed for 40 cycles in denature temperature 95 °C for 15 s and anneal temperature 60 °C for 1 min. Experiments were performed in triplicate for PEDF and glyceraldehyde-3-phosphate dehydrogenase (GAPDH). Primer sequences for *PEDF* were forward: 5′-CCA ACT TCG GCT ACG ATC-3′; reverse: 5′-GGC AGT AAC AGA GGC AAG-3′, and primer sequences for *GAPDH* were forward: 5′-TTC GAC AGT CAG CCG CAT CTT-3′; reverse: 5′-ATC CGT TGA CTC CGA CCT TCA-3′. Data were analyzed using *GAPDH* as the housekeeping gene control and the comparative CT (^∆∆^Ct) method for comparison of relative gene expression among the groups.

### Western blot assay

Two days after transfection, cells were washed with phosphate buffered saline (PBS), pH 7.4 (1×; GIBCO, Invitrogen) and collected by scraping. They were lysed in ice-cold Tris buffer (Invitrogen; 50 mM, pH 7.5) containing 5mM ethylenedinitrilotetraacetic acid (EDTA; Invitrogen), 300 mM Nacl, 0.1% Igepal, 0.5 mM NaF, 0.5 mM Na3VO4, 0.5 mM phenylmethane sulfonyl fluoride (PMSF), and antiprotease mixture (Roche Molecular Biochemicals, Invitrogen, Guangzhou, China), sonicated, and centrifuged at 13,000× g for 10 min. The supernatant was used for protein determination by the Bradford procedure (Bio-Rad, Xinhailing company, Shenzhen, China) and western blotting. The proteins were resolved on 12% sodium dodecyl sulfate polyacrylamide gels, transferred onto nitrocellulose membranes, and incubated with the appropriate antibodies. GAPDH was used as internal control. The anti-Vimentin antibody (sc-7558; Santa Cruz Biotechnology, Xinhailing company, Shenzhen, China) was used at a 1/400 dilution. The anti- αB-crystallin antibody (sc-22744; Santa Cruz Biotechnology, Xinhailing company) was used at a 1/400 dilution. The peroxidase-based detection was performed with Chemiluminescence Reagent (NEN Life Science, Xinhailing company, Shenzhen, China). According to the manufacturer’s instructions, sodium dodecyl sulfate polyacrylamide gels electrophoresis of samples at 110 V for 60 min, then transferred at 100 V for 150 min, then blocking for 60 min at room temperature, and finally incubated with primary antibody overnight at 4 °C and then with secondary antibody for 60 min at room temperature. We repeated each experiment three times.

## Results

### Primary human lens epithelial cell culture

**Figure 1:**
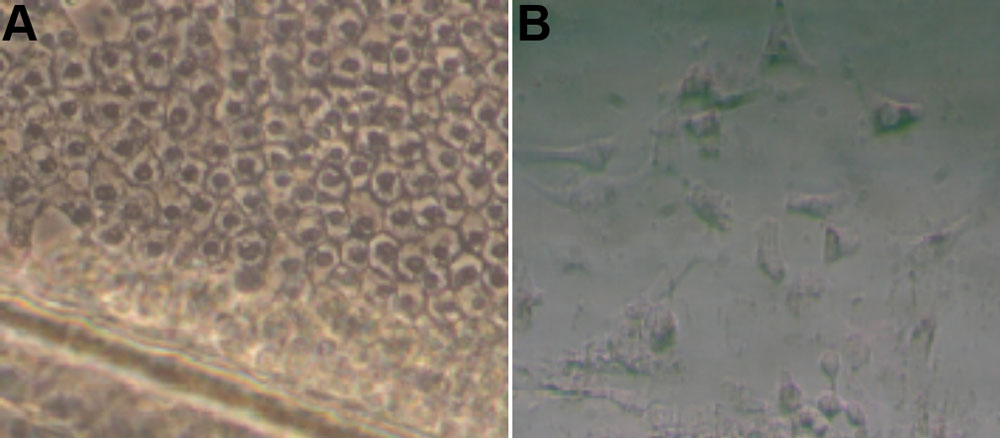
Images of human primary lens epithelial cells. Lens epithelial cells adhering to anterior capsule on the dish (A); 5 days later the human lens epithelial cells migrate from the anterior capsule to the dish (**B**).

Primary human lens epithelial cell cultures were established by the plating of intact capsules onto tissue culture dishes. At 24 h we observed healthy lens epithelium adherent to the capsule. By 5 days post plating, we found that epithelial cells had migrated from the capsule onto the dish. By 10 days of culture, the lens epithelium had expanded to 70%–80% confluence and possessed morphology consistent with the epithelial origin of the cells (Figure 1).

### Transduction of primary human lens epithelial cells with with small hairpin RNA (shRNA) lentiviral vectors

**Figure 2:**
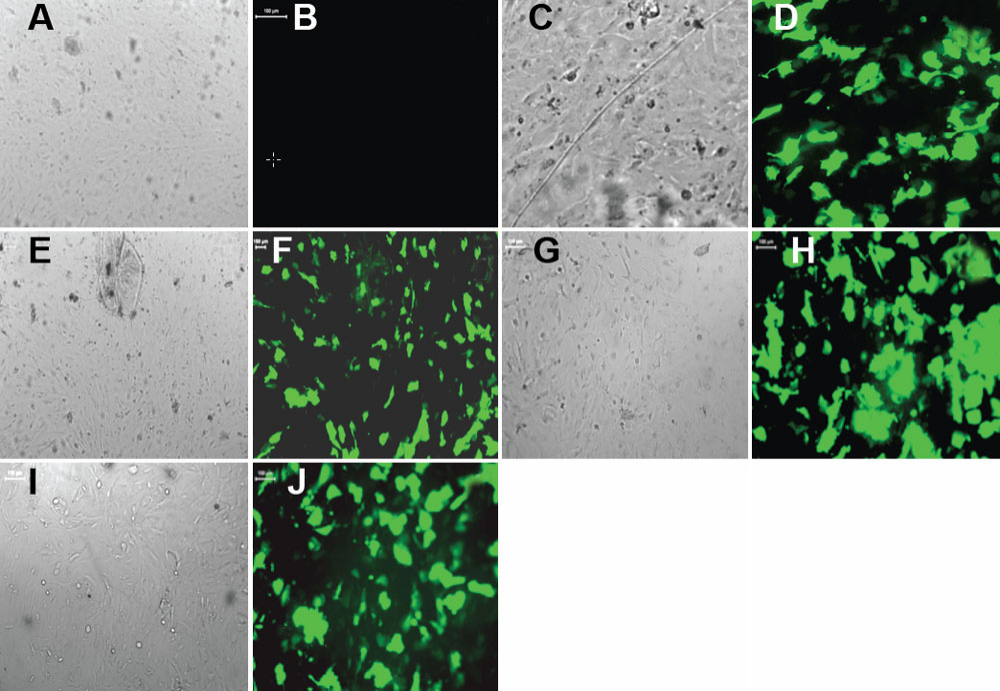
Images of human primary lens epithelial cells infected with different lentivirus. **A**, **C**, **E**, **G**, and **I** were taken under bright field. **B**, **D**, **F**, **H**, and **J** were taken under *green fluorescent protein* (*GFP*) field. **A** and **B** show the control group; the human primary lens epithelial cells were not infected in this group. **C** and **D** show group1 (mock group) infected with shRNA-scramble (random siRNA sequence). **E** and **F** show group 2, which is infected with shRNA1. **G** and **H** show group 3, which is infected with shRNA2. **I** and **J** show group 4, which is infected with shRNA3. The control group (**B**) has no GFP fluorescence, while almost 80–90% of the cells of group1, 2, 3, and 4 have green fluorescence.

Given the difficulty of gene transfer to primary epithelial cells using standard plasmid-based transfection techniques, we used lentiviral vectors to express shRNA constructs for the downregulation of *PEDF*. Co-expression of green fluorescent protein (GFP) with the shRNA constructs allowed for the evaluation of the efficiency of gene transfer to primary lens epithelium in culture. Epithelial cells were imaged via fluorescence microscopy 48 h after transduction with shRNA constructs, and we found that in each case approximately 80% of cells were transduced (Figure 2).

### Downregulation of pigment epithelium-derived factor (PEDF) expression by small hairpin RNA (shRNA) vectors

**Figure 3:**
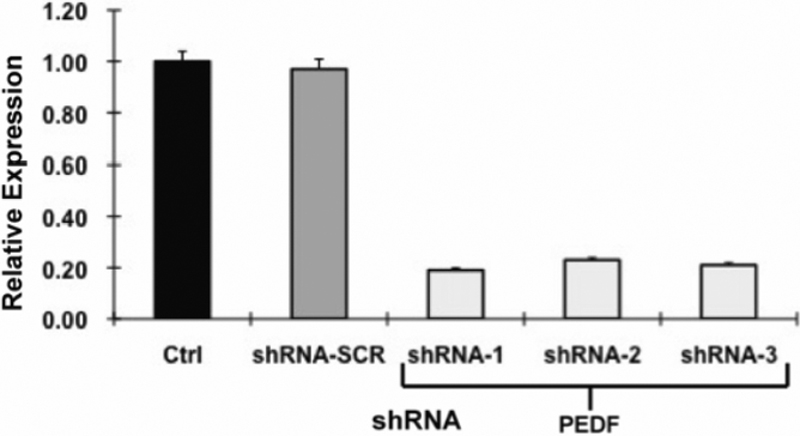
The relative RNA level of pigment epithelium-derived factor (PEDF) in human primary lens epithelial cells after RNA interference. Q-PCR was used to quantify the PEDF mRNA transcript 48 h after the infection. The relative PEDF mRNA expression level was reduced (p<0.01) in the sample 2 (shRNA1), sample 3 (shRNA2), and sample 4 (shRNA3) compared with control group. The mRNA transcripts were not significant differences (p>0.05) between control group and sample 1 (shRNA1 with random RNA sequence). The PEDF mRNA was significantly down regulated with RNAi in group 2, 3, and 4. We used One-way analysis of variance (ANOVA); each experiment was repeated 3 times (n=3).

We next analyzed the ability for shRNA directed against *PEDF* to downregulate *PEDF* expression. After 48 h from transduction with shRNA expressing lentiviral constructs, primary lens epithelial cells were harvested and *PEDF* mRNA expression determined by real time quantitative polymerase chain reaction (qPCR). Nontransfected cells and cells transfected with nonspecific scrambled shRNA vector had identical *PEDF* expression. All three *PEDF* shRNAs significantly decreased (p<0.01) the expression of *PEDF* in the primary epithelial cultures compared to control and shRNA-scramble groups (Figure 3).

### Vimentin and αB-crystallin expression are modulated by small hairpin RNA (shRNA) directed against pigment epithelium-derived factor (*PEDF*)

**Figure 4:**
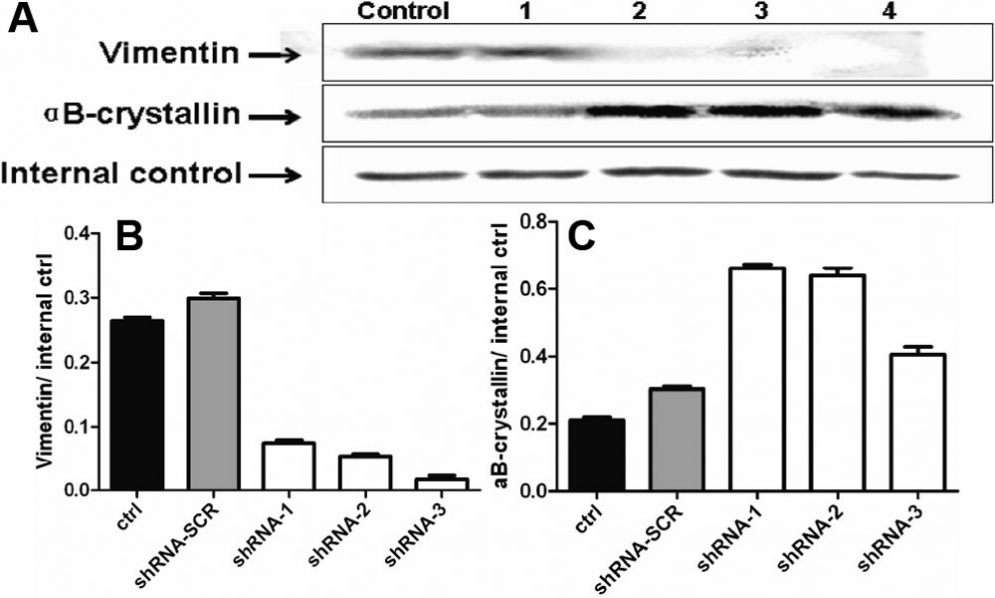
The relative levels of vimentin and αB-crystallin in Human primary lens epithelial cells after RNA interference (RNAi), measured by western blot. The internal control is Glyceraldehyde 3-phosphate dehydrogenase (GAPDH; A). The expression of vimentin in different groups of human primary epithelial lens cells measured 48 h after lentiviral infection. The levels of Vimentin in the control group are considerably higher compared to shRNA-1, 2, 3 groups (p<0.05). There are no significant differences between the levels of Vimentin detected in the control group and the shRNA-scramble group (p>0.05, shRNA-scr with random RNA sequence). One-way analysis of variance (ANOVA) were applied to each treatment (n=3), and the standard error of the mean (SEM) was used in the graph (B). The expression of αB-crystallin in different groups of human primary epithelial lens cells measured 48 h after lentiviral infection. The levels of αB-crystallin in the control group were considerably lower compared to shRNA-1, 2, 3 groups (p<0.05). There were no significant differences between the levels of αB-crystallin detected in the control group and the shRNA-scramble group (p>0.05, shRNA-scr with random RNA sequence). One-way analysis of variance (ANOVA) was applied to each treatment (n=3),and the standard error of the mean (SEM) was used in the graph (C).

Given the known changes in *PEDF* expression in catarctous lenses, we next looked at the changes in key lens structural proteins implicated in the formation of cataract following the downregulation of *PEDF* expression by shRNA. Western blotting was used to determine the concomitant effects of *PEDF*-directed shRNA on the expression of vimentin and αB-crystallin. Figure 4 shows the distribution of vimentin and αB-crystallin and the analysis of protein expression level in the different groups 48 h after infection. Compared with the nontransfected group, αB-crystallin expression was about 30% higher in the shRNA-scramble group, about 219% higher in the shRNA-1 group, about 204% higher in the shRNA-2 group, and about 93% higher in the shRNA-3 group. Vimentin expression was about 13% lower in the shRNA-scramble group, about 72% lower in the shRNA-1 group, about 79% lower in the shRNA-2 group, and about 93% lower in the shRNA-3 group. The results indicate that shRNA 2, shRNA3, and shRNA4 RNAi can significantly downregulate the expression of vimentin and upregulate the expression of αB-crystallin in human primary lens epithelial cells (p<0.05; one-way analysis of variance [ANOVA] n=3).

## Discussion

*PEDF* is expressed virtually in all ocular tissues of the human eye [1]. Most studies on this gene in eye tissue have focused on the retinal and ciliary body [1]. Information is known on its neuroprotective and anti-angiogenic actions in the eye [28], and there is some association between *PEDF* and age-related eye diseases, such as age-related macular degeneration [29]. There have also been studies of this gene on the lens, but most research has focused on location and expression level. The novelty of our experiment is that we associate *PEDF* with lens proteins. Since all these proteins are affected by the age-related factors, there may be some associations between them and *PEDF*. Through the downregulation of *PEDF* with the RNAi technique, we obtained two major findings: (1) the protein level of αB-crystallin is significantly higher than the control group; and (2) the protein level of vimentin is significantly lower than the control group. We do find the associations among *PEDF* down regulation and the expression of αB-crystallin and vimentin even through it’s hard to tell if the down-regulation of *PEDF* is the direct or the unique factor for the changes of lens protein αB-crystallin and vimentin.

First, in this experiment, primary human lens epithelial cells were used. Their physiological characteristics are much more similar as in vivo than human lens epithelial cell line. Since the primary cells are very hard to be transfected, we performed the PEDF RNAi with the help of lentivirus infection. To improve the success rate, we designed three different primers. According to the Real time quantitative Polymerase Chain Reaction (qPCR) results, all of the experiment groups have high RNAi efficiency.

Second, in this experiment we realized the downregulation of *PEDF* artificially. We removed the other contributing factors, such as age-related factors and homeostasis factors, so that we could focus on the association between *PEDF* and the lens proteins. We also set up the shRNA-scramble control group, which helped us prove that the changes to the lens protein were not caused by infection reagents or some other experimental factors other than the downregulation of *PEDF*.

*PEDF* was strongly downregulated (by 5.9-fold) in senile cataract [9], and we found that downregulation of *PEDF* can cause the higher expression of αB-crystallin. Therefore, we suggest that the expression of αB-crystallin is higher in senile cataract, which is consistent with two previously reported findings: 1) the reduced thermal stability and the dominant negative effects of mutant αB-crystallin may be the direct cause of cataract because αB-crystallin null mice have clear lenses [24]; and 2) the decrease of vimentin may be initiated by damage to the epithelial cells, leading to degradation of the cytoskeleton, and appears to be related to the formation of age-related cataract [27].

Oxidative damage to lens proteins is a major factor leading to cataract formation. Idiopathic senile, diabetic, and myopic cataractogenesis appear to be dependent on oxidative damage to lens proteins [30]. Another study indicated that the amount of inhibitory *PEDF* produced by retinal cells was positively correlated with oxygen concentrations [31]. We therefore suggest that the change in vimentin and αB-crystallin may be caused by downregulation of *PEDF*, which is connected to oxidative cellular stress.

α-Crystallins, especially αB-crystallin, are expressed in several other tissues under stress conditions. α-Crystallin appears to function as a molecular chaperone in prevention of stress-induced precipitation of β- and λ-crystallins [32]. αB-crystallin is a small heat shock protein so it can be constitutively expressed and increased in response to cellular stresses [33], while cellular stresses can also reduce expression of nonessential genes. We therefore hypothesize that the upregulation of αB-crystallin and the downregulation of vimentin caused by downregulation of *PEDF* were due to a cellular stress response. If this is the case, we can also investigate whether the other stress response proteins can be increased upon *PEDF* downregulation.

Because crystallins, proteins related to the small heat shock protein family, have been shown to bind to vimentin and inhibit filament assembly [34], it is still unknown whether the expression change of the αB-crystallin was induced by *PEDF* downregulation or induced by the change in vimentin expression. Therefore, our future work will focus on the fundamental mechanism causing the change of the lens proteins caused by the downregulation of *PEDF*.

In summary, this study demonstrated a significant overexpression of αB-crystallin and downexpression of vimentin in human primary lens epithelial cells through RNAi to *PEDF*. Since vimentin and aB-crystallin are two important proteins of human lens and are changed in the process of cataract formation, there may be some relationship between the downregulation of *PEDF* and cataract.
